# How to Choose the Right Treatment for Membranous Nephropathy

**DOI:** 10.3390/medicina59111997

**Published:** 2023-11-14

**Authors:** Luigi Peritore, Vincenzo Labbozzetta, Veronica Maressa, Chiara Casuscelli, Giovanni Conti, Guido Gembillo, Domenico Santoro

**Affiliations:** 1 Unit of Nephrology and Dialysis, Department of Clinical and Experimental Medicine, University of Messina, 98125 Messina, Italy; vincenzo.labbozzetta@gmail.com (V.L.); veronicamaressa@virgilio.it (V.M.); chiara.casuscelli88@gmail.com (C.C.); 2 Pediatric Nephrology Unit, AOU Policlinic “G Martino”, University of Messina, 98125 Messina, Italy; giovanniconti@hotmail.com

**Keywords:** membranous nephropathy, immunosuppressive therapy, glomerulonephritis, nephrotic syndrome, rituximab, belimumab, bortezomib, complement children, pregnancy, renal transplant

## Abstract

Membranous nephropathy is an autoimmune disease affecting the glomeruli and is one of the most common causes of nephrotic syndrome. In the absence of any therapy, 35% of patients develop end-stage renal disease. The discovery of autoantibodies such as phospholipase A2 receptor 1, antithrombospondin and neural epidermal growth factor-like 1 protein has greatly helped us to understand the pathogenesis and enable the diagnosis of this disease and to guide its treatment. Depending on the complications of nephrotic syndrome, patients with this disease receive supportive treatment with diuretics, ACE inhibitors or angiotensin-receptor blockers, lipid-lowering agents and anticoagulants. After assessing the risk of progression of end-stage renal disease, patients receive immunosuppressive therapy with various drugs such as cyclophosphamide, steroids, calcineurin inhibitors or rituximab. Since immunosuppressive drugs can cause life-threatening side effects and up to 30% of patients do not respond to therapy, new therapeutic approaches with drugs such as adrenocorticotropic hormone, belimumab, anti-plasma cell antibodies or complement-guided drugs are currently being tested. However, special attention needs to be paid to the choice of therapy in secondary forms or in specific clinical contexts such as membranous disease in children, pregnant women and patients undergoing kidney transplantation.

## 1. Introduction

Membranous nephropathy (MN) is an autoimmune disorder affecting the kidney glomerulus and is one of the most prevalent diseases causing nephrotic syndrome (NS) in adults [1]. Its prevalence has changed over years, leading it to become the pre-eminent pathological finding in China between 2015 and 2019 [2] and accounting for 15% of kidney biopsies performed in Singapore between 2008 and 2018 [3], while a decreasing trend was observed in United States of America, United Kingdom and Italy during the last decade. Clinically, MN can range from an asymptomatic proteinuria, which occurs in about one-third of patients, to full-blown NS, manifested by the remaining two-thirds. The latter is a condition defined by a characteristic pentad that includes proteinuria of >3.5 g per 24 h, hypoalbuminemia, edema, hypercholesterolemia and lipiduria; less frequent manifestations are hypertension and thromboembolic events. In the absence of other conditions, kidney impairment at the time of diagnosis is not frequent [4]. In the absence of any immunosuppressive therapies, the prognosis can be variable. Five years after diagnosis, up to 30% of patients may achieve complete remission, defined as proteinuria below 200 mg in 24 h, while up to 40% of patients may show partial remission, with proteinuria below 2.000 mg in 24 h [5]. After 10 years, the rate of patients developing kidney failure is 35%; however, among patients who do not develop NS during this period, only 2% reach end-stage renal disease (ESRD) [6].

The 2021 Kidney Disease Improving Global Outcomes (KDIGO) guidelines recommend the use of rituximab (RTX) or a calcineurin inhibitor (CNI) as the initial treatment for patients at moderate risk of disease progression, while the use of first-line cyclophosphamide (CYC) is recommended for high-risk patients. Conversely, patients at low risk of progression do not require immunosuppressive therapy and should be treated only with support care [7]. However, up to 30% of patients fail to respond to standard therapy. Some patients develop drug intolerance or serious adverse effects, especially infections. Also, relapses are common. This has led to the search for more specific and better-tolerated immunomodulatory drugs that improve long-term outcomes. New therapies directly targeting B cells, plasma cells and antibody production have shown encouraging results, especially in patients with poor tolerance or who are refractory to conventional treatments. In this review, we will describe in detail the therapeutic options for the treatment of membranous glomerulonephritis, starting from the supportive treatment approach through to the use of diuretics and antiproteinuric drugs, treatment of the nephrotic syndrome, traditional immunosuppressive therapy and new patterns of immunosuppression. We will also address the treatment of secondary membranous glomerulonephritis and the treatment in particular conditions such as pregnancy and renal transplantation.

## 2. Membranous Nephropathy: Pathophysiology and Autoantibodies

MN is an immunocomplex disease. Pathogenesis begins with the binding of circulating antibodies to antigens present on podocytes. In the secondary forms, however, the initiating trigger could be the deposition of immune complexes in the subepithelial space [8].

**Figure 1:**
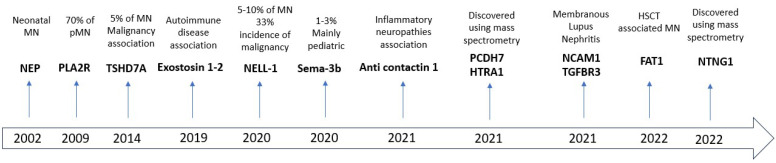
History of discovery of various antigens. FAT1, protocadherin FAT1; HTRA1, serine protease HTRA1; MN, membranous nephropathy; NCAM1, neural cell adhesion molecule 1; NEP, neutral endopeptidase; NELL, neural-tissue-encoding protein with EGF-like repeats; NTNG1, netrin G1; PCDH7, protocadherin 7; PLA2R, phospholipase A2 receptor 1; pMN, primary membranous nephropathy; TGFBR3, transforming growth factor beta receptor 3; TSHD7A, thrombospondin.

The first research on the pathogenesis of the disease dates back to 1980, from studies conducted on mouse models of Heymann’s nephritis; however, the antigen responsible for this disease, megalin, was not found to be present in humans. The first data in humans were found in 2002 following a case of neonatal MN, in which the mother, who had been immunized during a previous pregnancy, had transferred the antibodies directed against neutral endopeptidase (NEP) to the newborn. In 2009, the discovery of antigens directed against phospholipase A2 receptor 1 (PLA2R1) represented a milestone in understanding the pathogenesis responsible for about 70% of adult MN cases. In 2014, a second antigen, thrombospondin, was identified [9]. Recently discovered antigens include exostosin 1 and 2 (associated with ANA, dsDNA and anti Ro/LA and present in 50% of class V lupus nephritis) [10] and NELL (neural-tissue-encoding protein with EGF-like repeats)-1 [11]. In addition, autoantibodies directed against contactin have been found in patients with inflammatory neuropathy and MN; although its detection cannot be used to diagnose the disease, its levels correlate with the clinical course [12]. In the pediatric population, semaphorin 3B was also recently discovered as a new antigen; its prevalence ranges from 1 to 3% among all MN in the adult population but is more common in pediatric patients, accounting for 15% of all MN cases; moreover, its detection is more common in the case of a positive familiar history [13]. In membranous lupus nephritis, neural cell adhesion molecule 1 (NCAM1) [14] and transforming growth factor beta receptor 3 (TGFBR3) [15] were discovered as new antigens, while protocadherin FAT1 [16] was associated with hematopoietic stem cell transplant (HSCT)-MN. If these three last antigens are suspected due to their association with LES or HSCT, immunohistochemistry/immunofluorescence can be performed to obtain additional prognostic clues. Finally, protocadherin 7 (PCDH7) (associated with prostate carcinoma but with a favorable prognosis) [17], serine protease HTRA1 (4% of pMNs) [18] and netrin G1 (NTNG1), whose diagnostic and clinical role still needs to be defined [19], were identified over the past two years as new target antigens using mass spectrometry. Table 1 shows the different antigens and their features. Figure 1 shows the history of discovery of the various antigens.

PLA2R is a transmembrane receptor of the mannose receptor family whose role in human cells is still unknown. Anti-PLA2R antibodies, which have a prevalence of 70–80% in primary MN, have diagnostic, prognostic and predictive value. Positivity to these antibodies demonstrated a sensitivity of 78% and a specificity of 99% in the diagnosis of NM; moreover, the specificity reaches 100% when the search for PLA2R in serum is complemented by staining of the kidney biopsy. These discoveries made it possible to diagnose MN even in those situations where a biopsy is complicated, such as in patients with a single kidney or with an increased risk of bleeding. Anti-PLA2R antibodies are able to anticipate the disappearance of proteinuria in response to therapy by several months and, conversely, in disease relapses. Finally, higher antibody titers correlate with a worse prognosis and a slower response to therapy [20]. As proved by Hink et al. [21] in a single-arm prospective cohort study including 65 patients with PLA2Rab-biopsy-proven MN, regular monitoring of such antibodies allowed the design of a tailor-made therapy according to the level of PLA2R antibodies. Oral cyclophosphamide (1.5 mg/kg/day) plus intravenous methylprednisolone (1000 mg for 3 days at days 1–3) and subsequent oral prednisone (0.5 mg/kg/day) were administered for 8 weeks; if detection of the antibodies subsequently became negative, CYC was stopped and the steroids tapered off; alternatively, if no disappearance of antibodies was observed, therapy was re-administered for another 8 weeks for up to two more times for a maximum of 24 weeks of CYC therapy. If no remission was reached after 24 weeks, patients were shifted to MMF + steroids therapy and CYC was withdrawn. Notably, patients treated with this serology-guided protocol received a lower cumulative dose of CYC compared with patients treated without antibody monitoring (11.8 g vs. 18.9 g) [22].

Thrombospondin-1-domain-containing 7 A (THSD7A) is a transmembrane protein localized in podocytes. The prevalence of antithrombospondin antibodies is up to 5% of all NM cases and up to 10% of cases without PLA2R antibodies. Several studies have reported an association with malignancy; therefore, more intensive malignancy screening is required in these patients. In addition, the presence of these antibodies in tumor cells has been described as well as, more importantly, a reduction in antibody titer and proteinuria following chemotherapy [20]. Like PLA2R antibodies, the antithrombospondin antibody titer can anticipate both clinical remission and clinical relapse by several months. However, according to KDIGO 2021, at the current time, there is no sufficient data to perform a diagnosis of MN only through anti-THSD7A antibody detection.

NELL (neural-tissue-encoding protein with EGF-like repeats)-1, a glycoprotein poorly expressed in adult tissues but essential for ossification during child growth [23], is the second most prevalent auto-antigen recently discovered. It accounts for a prevalence of MN ranging from 5 to 10%, including in both primary and secondary forms of disease. Among the latter, NELL-1 MN has been associated with malignancy, drugs, autoimmune disease, transplant, hepatitis and sarcoidosis. Of note, one study reported an incidence of malignancy of up to 33% [23]; consequently, detection of NELL-1 should warrant more exhaustive research for secondary causes [24].

However, how these autoantibodies are formed is still largely unknown. Genetics plays a major role, and an association with a single nucleotide polymorphism at the HLA-DQA1 locus has been described. Chinese studies that have found a different prevalence between rural and industrialized areas also highlight that environmental pollution may play a role. The development of such autoantigens can likely be attributed to the loss of peripheral or central tolerance, altered expression of antigens or molecular mimicry mechanisms [25]. Nonetheless, the binding of such antibodies to the corresponding antigens or the deposition of the immunocomplex leads to renal damage through both complement-dependent and -independent mechanisms [25]. Theoretically, the presence of IgG subclasses that can activate complement more strongly, such as IgG3, could correlate with a poorer prognosis, whereas the presence of IgG4, which is considered a weak complement activator, could be a positive prognostic factor. However, clues supporting such hypotheses are lacking [8].

## 3. An Open Question: Is Biopsy Still Necessary in Patient with PLA2R Antibodies?

According to the latest KDIGO guidelines, patients with NS who test positive for anti-PLA2R antibodies do not require a biopsy to diagnose the disease; however, in some specific cases, it can be considered. Data on anti-THS7DA are insufficient to extend the same recommendation to this antibody [7].

However, a kidney biopsy may be performed for purposes other than diagnosis alone. A Mayo Clinic retrospective study covering the period from 2015 to 2018 analyzed 97 patients with primary MN who were positive for PLA2R antibodies but still underwent biopsy. Of these, 60 had an eGFR greater than 60 mL/min, and renal biopsy did not alter the therapy or patient management in any way, even in the two cases diagnosed with superimposed diabetic nephropathy or focal and segmental glomerulosclerosis (FSGS). On the other hand, the discovery of additional findings, such as acute interstitial nephritis and crescents in the group of patients with an eGFR below 60 mL/min, actually had an impact on therapy, suggesting more aggressive therapy and additional vasculitis antibody testing [26]. Also, the discovery of coexisting diabetic nephropathy could explain the persistence of proteinuria during follow-up despite the disappearance of PLA2R antibodies.

Similarly, Wiech et al. analyzed 263 patients with a histological diagnosis of MN, including 194 who were positive for anti-PLA2R antibodies. Of the latter, twelve (6%) received an additional diagnosis such as diabetic nephropathy (*n* = 5), interstitial nephritis (*n* = 5) and IgA nephropathy (IgAN) (*n* = 2). Of note, patients with an additional diagnosis were those with the highest creatinine and lowest eGFR values. Interestingly, the proportion of patients with an additional diagnosis other than MN was slightly higher in patients who were negative for anti-PLA2R antibodies [27].

In any case, the KDIGO guidelines recommend that a kidney biopsy be performed in the case of an unusual clinical course, such as a rapid decrease in the estimated glomerular filtration rate (eGFR), serological abnormalities or when patients do not respond to immunosuppressive therapy and show a progressive decrease in eGFR or persistent NS despite the disappearance of PLA2R antibodies. In any case, the possibility of a renal biopsy must always be assessed on a case-by-case basis, taking into account both the costs and the risks to the patient [7].

## 4. Membranous Nephropathy Supportive Therapy

**Figure 2:**
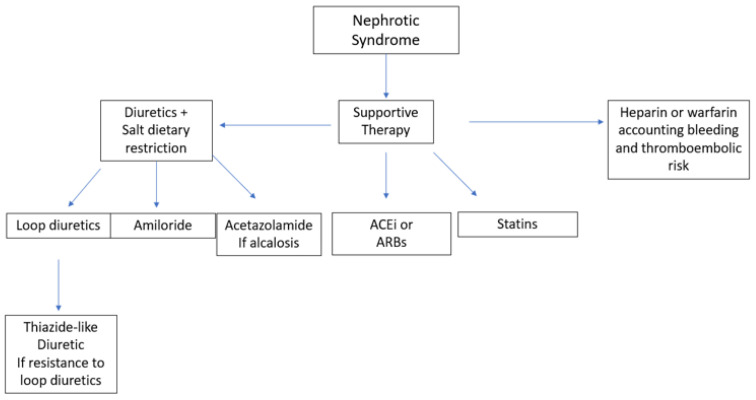
Different therapeutic options for the symptomatic treatment of MN. ACEi, angiotensin-converting enzyme (ACE) inhibitors; ARB, angiotensin-receptor blockers.

Regardless of the degree of proteinuria, renal function and extent of NS, all patients with MN should receive the best possible supportive care and be treated to prevent possible complications from the disease [7]. Such treatment may reduce both the morbidity and mortality independently of immunosuppressive therapy. Figure 2 shows different therapeutic options for the symptomatic treatment of nephrotic syndrome.

The treatment of edema includes both the use of diuretics and dietary salt restriction. Loop diuretics administered twice daily are the first-line therapy [28]. As the prolonged administration of furosemide can lead to adaptation mechanisms, there is some evidence of better results with torsemide and bumetanide [29]. If there is superimposed diuretic resistance, a thiazide-like diuretic such as chlortalidone, hydrochlorothiazide or metolazone can be added to prevent sodium reabsorption in the distal areas of the nephron. While using such a combination of diuretics, administration of a thiazide-like diuretic two to five hours before a loop diuretic can minimize distal sodium reabsorption. The use of amiloride and acetazolamide may help in the management of hypokalemia and metabolic alkalosis, respectively. As gastrointestinal absorption of these diuretics may be affected by bowel wall edema, intravenous loop diuretics may be a valid alternative. Intravenous albumin may help to improve the delivery of the diuretic to its target site in the nephron and should be considered if serum albumin levels are below 2.0 g/dL [30]. Daily sodium intake should not exceed 2 g or 88 mEq, while water restriction is not required unless hyponatremia or fluid overload is present [7].

Angiotensin-converting enzyme inhibitors (ACEi) or angiotensin-II-receptor blockers (ARBs) are the first-line therapy for blood-pressure control thanks to their additional effect in reducing proteinuria. Reaching the target blood pressure (i.e., systolic blood pressure <120 mmHg) can both protect against cardiovascular risks and slow down the loss of the GFR. On the other hand, loss of renal function can be prevented if proteinuria is reduced to less than 0.5 g per day or slowed to less than 1.5 g per day [7]. In addition, the reduction in proteinuria and subsequent increase in serum protein and albumin levels can prevent infection, metabolic and thromboembolism risk. ACEi and ARBs can reduce proteinuria by up to 50%. These drugs should be given at the maximum tolerated dose and should only be discontinued if there is an increase in creatinine of more than 30%, if there is a continuous loss of renal function, or if the induced hyperkalemia no longer responds to any available drug treatment. If this is the case, a direct renin inhibitor (DRI) or a mineralocorticoid receptor antagonist (MRA) may be used to replace ACEi or ARBs [31] but be an addition to them [32]. Finally, non-dihydropyridine calcium-channel blockers (CCB) [33] may also reduce proteinuria, though only in a minor way.

Hyperlipidemia must be treated if the patient has other risk factors for cardiovascular disease, including diabetes, smoking, hypertension or being overweight. The first step in treating lipid abnormalities is diet and lifestyle modification. Statins are effective in lowering lipid levels, and there is some evidence that atorvastatin may also reduce proteinuria [34] compared with rosuvastatin. Currently, there are insufficient data to broadly use second-line therapies such as ezetimibe [35] or PCSK9 inhibitors [7].

Among the different forms of glomerulonephritis, MN is the one that carries the greatest risk of thromboembolic events, especially deep-vein thrombosis and renal-vein thrombosis [36], and the risk is even higher depending on the degree of proteinuria and if albumin levels fall below 2.5 g/L. Full anticoagulation is mandatory if a thromboembolic event has already occurred. On the other hand, prophylactic anticoagulation should be carefully evaluated to account for both the risk of bleeding and thromboembolism [7]. The first-line therapy is heparin (or a derivative thereof) or warfarin; further studies are currently needed to investigate the potential role of direct oral anticoagulants. In patients with an albumin level below 2.5 g/L, a high venous thromboembolism risk and a high bleeding risk that complicates the use of warfarin, aspirin can be used, as it is in patients at risk of arterial thromboembolism [37].

## 5. History of Immunosuppressive Therapy in Membranous Nephropathy

The first drugs used to treat MN were exclusively glucocorticoids, although trials conducted with prednisone showed no or only transient benefit [38,39]. In addition, a trial involving cyclophosphamide failed to show any improvement in proteinuria or renal function [40], and retrospective studies that included chlorambucil raised suspicions that it might be associated with cancer [41].

In 1984, a multicenter Italian RCT assigned 67 patients with MN to symptomatic treatment or to an alternating combination of chlorambucil and glucocorticoids for six months. At months 1, 3 and 5, 1 g of intravenous methylprednisolone was administered for 3 days and 0.5 mg/kg oral prednisone for a subsequent 27 days, while chlorambucil at 0.2 mg/kg/day was given at months 2, 4 and 6. The treatment group showed a stabilization of renal function and an improvement in proteinuria, and these results were confirmed 10 years later [42]. This approach, also known as “Protocollo Ponticelli”, was shown to achieve better results than glucocorticoid administration alone [43]. In 1998, results were published from a trial designed to prove the non-inferiority of cyclophosphamide compared to chlorambucil in alternating therapy; patients treated with the former had better rates of complete and partial remission, relapsed less frequently and also seemed to tolerate the drug. Since then, cyclophosphamide has replaced chlorambucil in the approach known as the “Modified Ponticelli” [44].

Early indications of a potential role for cyclosporine in the treatment of MN came from several observational studies in which patients achieved partial or complete remission [45,46,47]. However, this treatment option was hampered by frequent relapses upon the reduction or withdrawal of the drug and by its potential nephrotoxicity. Subsequently, two randomized studies showed an improvement in slowing the decline of renal function and proteinuria compared with the placebo [48] and better remission rates when combined with prednisone compared with placebo plus prednisone [49]. Similar to cyclosporine, tacrolimus also showed a good response but had a high relapse rate upon discontinuation. These data come from retrospective [50], intervention [51] and randomized studies [52]. When tacrolimus was used in combination with glucocorticoids, better results in terms of remission rates and relapses were obtained with 24 months of therapy compared with 12 months [53]. Another proposed drug was mycophenolate mofetil (MMF), although this had a higher risk of relapse compared with cyclophosphamide [54] and a lower rate of complete–partial remission when combined with glucocorticoids compared with cyclophosphamide [55]. It should be noted that no difference in terms of proteinuria reduction or remission achievement was found between the use of MMF and supportive therapy alone [56].

## 6. Treatment of Membranous Nephropathy in KDIGO 2021

**Figure 3:**
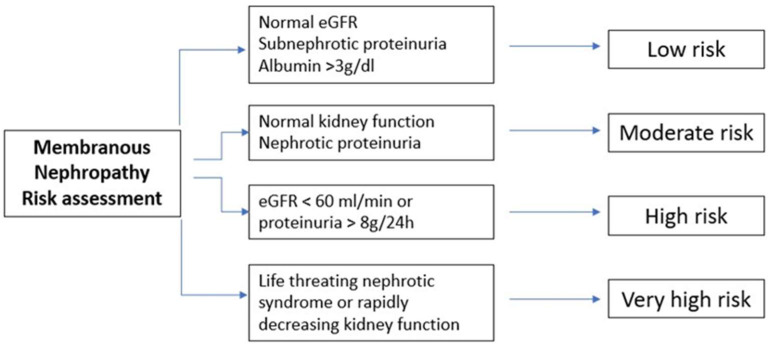
Schematic representation of different subgroups with different risk levels of progression toward end-stage renal disease. eGFR, estimated glomerular filtration rate.

Since patients suffering from MN can spontaneously experience remission, it is crucial to determine which patient would benefit more from immunosuppressive therapy and who can be treated only with supportive therapy. Hence, the latest KDIGO guidelines [7] suggest assessing the risk of the loss of kidney function by dividing patients into subgroups of low, moderate, high and very-high risk of progression toward end-stage renal disease, accounting for both clinical and laboratory criteria. Figure 3 summarizes the four different subgroups with regard to their risk of progression toward end-stage renal disease.

Consequently, it is suggested to wait 6 months while using maximal anti-proteinuria therapy since spontaneous remission can occur. On the other hand, if high-level proteinuria, high-tier PLA2R auto-antibodies or low-molecular-weight proteinuria are present, an earlier re-evaluation is mandatory, while if kidney function is rapidly decreasing and if nephrotic syndrome is unresponsive to symptomatic therapy, an early course of immunosuppressive therapy should be started at soon as possible. The chance of spontaneous remission is higher in patients with proteinuria below 4 g/day compared with those below 8 g/day and 12 g/day (45% vs. 34% vs. 25–40, respectively) and in patients with lower tiers of anti-PLA2R antibodies compared with higher ones. Moreover, in addition to single values, it is fundamental to evaluate the trajectory of such parameters.

Consequently, immunosuppressive therapy should be practiced only in patients at risk of progressive kidney injury. It is not required if proteinuria is not in nephrotic range (i.e., below 3.5 g/day), if serum albumin is greater than 30 g/L and if the eGFR is above 60 mL/min. Such patients usually have low risk of thromboembolic complications and have few or any symptoms and can therefore be managed only with conservative therapy. Immunosuppressive therapy in such patients would add risks without any potential benefits.

**Figure 4:**
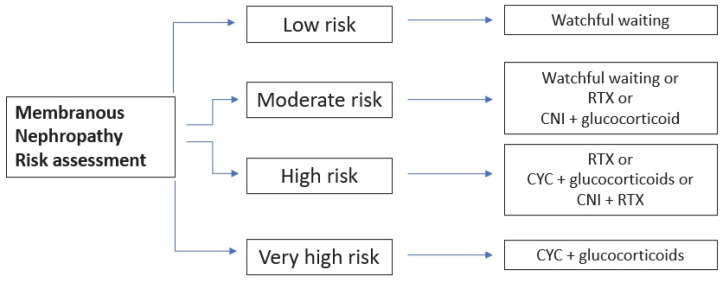
Schematic representation of different subgroups with respective therapy. CYC, cyclophosphamide; CNI, calcineurin inhibitors; RTX, rituximab.

On the other hand, if there is at least one risk factor for disease progression, immunosuppressive therapy is recommended. The choice of the specific therapy should account for the patients’ characteristics, drug availability, preference from patients and physicians and the specific side effect of each drug. Figure 4 summarizes the choice of therapies, accounting for the risk category. Different therapeutical protocols are represented in Table 2.

The use of MMF is not included since KDIGO 2012 argued against its utility. Many randomized control trials (RCTs) and cohort studies proved that rituximab (RTX) and tacrolimus/cyclosporine can increase the rate of both complete and partial remissions, with a better safety profile than cyclophosphamide. However, the use of calcineurin inhibitors (CNIs) is hampered by their high relapse rate and, consequently, they can be used in monotherapy only in the case of a moderate risk of progression. Cyclophosphamide (CYC), on the other hand, reduces the risk of kidney failure to a greater extent, but it should be used only in high-risk patients due to its toxicity burden. The cumulative dose of the latter should not exceed 36 g in order to avoid malignancy or 10 g to preserve fertility.

The definition of relapsing MN varies among different authors. While some authors define relapse as an increase in proteinuria above 3.5 g/d, the working group of KDIGO 2021 defined a relapse as an increase in proteinuria with a coexistent decrease in serum albumin. If a patient experiences a relapse after a first course of rituximab, a second course of the drug can be administered. On the other hand, if a relapse occurs after a course of CNI + steroid, rituximab can be added alone or in combination with CNI. Finally, if the first-line therapy consisted of cyclophosphamide + glucocorticoids, a second course of the same schedule can be repeated, accounting for the maximum dose tolerated, or, alternatively, CNI + rituximab or rituximab alone may represent a second-line therapy.

There is no consensus, on the other hand, about the proper definition of resistant disease. If anti-PLA2R antibodies are detectable before first-line therapy, the disease can be defined as resistant if the antibody titer is still high after one course of therapy. Conversely, proteinuria evaluation cannot be used since it can persist for up to 24 months after therapy. If patients are not PLA2R-positive, persistence of the nephrotic syndrome can define a resistant disease. In evaluating resistant disease, the first step consists in checking the adherence to therapy by measuring the levels of B cells, anti-rituximab antibodies, CNI and IgG or the development of leukocytopenia in patients using cyclophosphamide.

If the first line-line therapy consists of RTX, CNI can be added to the therapy if the eGFR is stable. If the eGFR is decreasing or if even CNI + RTX fails to achieve a response, a trial of cyclophosphamide + glucocorticoids can be made. On the other hand, if CNI represents the first attempt, RTX can be administered if the kidney function is stable; however, if it is unstable or if RTX did not achieve a response, an alkylating agent must be tried. If the latter represents the first-line therapy, RTX can be added before trying another course of alkylating agents. If patients fail to respond to either RTX or CYC, their enrolment in trials with experimental drugs is suggested (see “Novel therapeutic approaches for Membranous Nephropathy” below).

In our center, intravenous methylprednisolone is usually administered in an inpatient setting, while RTX can be administered to both outpatients and inpatients.

## 7. Rituximab

The last drug included in KDIGO for the treatment of MN was RTX, a chimeric IgG1 monoclonal antibody that exerts its B-cell depleting effect through its binding to CD20. To date, numerous studies have been conducted to evaluate the efficacy of this treatment in monotherapy and in combination with the drugs already in use. The GEMRITUX randomized trial, published in 2017, evaluated 6 months of non-immunosuppressive drug therapy (NIAT) in combination with 375 mg/m^2^ RTX administered intravenously on days 1 and 8 (*n* = 37) or non-immunosuppressive therapy alone (*n* = 38). After 6 months, 13 patients in the NIAT-RTX group and 8 patients in the NIAT group achieved remission. At the prolonged follow-up, with a median follow-up of 17 months, the difference was significant, with remission occurring in 64.9% of patients in the NIAT-RTX group and only 34.2% in the NIAT group (*p* < 0.01). In addition, PLA2R Ab-depletion rates in the NIAT-RTX and NIAT groups at 3 months were 56.0% and 4.3%, respectively (*p* < 0.001) [57]. The different combination therapies were then compared to determine which patient profile was best suited to treatment with a specific therapeutic protocol. STARMEN, a study performed in Spain and published in 2020, compared six months of cyclic treatment with corticosteroids and cyclophosphamide with sequential treatment using tacrolimus (full dose for six months and tapering during the following three months) and RTX (1 g monthly for six months). A total of 86 patients with primary MN were randomized into two subgroups of 43 patients. Out of these, 83.7% of patients (*n* = 36) achieved complete or partial remission in the corticosteroid–cyclophosphamide subgroup and 58.1% (*n* = 25 patients) in the tacrolimus-RTX subgroup, with complete remission after two years in 60% of patients (*n* = 26 patients) in the corticosteroid–cyclophosphamide subgroup and in 26% (*n* = 11 patients) of the tacrolimus-RTX subgroup. The rate of patients achieving an immunological response, i.e., a disappearance of anti-PLA2R antibodies, was significantly higher in the corticosteroid–cyclophosphamide group than in patients receiving tacrolimus and rituximab, both at three months (77% vs. 45%) and six months (92% vs. 70%). Relapsing disease was observed in three patients in the tacrolimus-RTX subgroup and only in one patient in the corticosteroid–cyclophosphamide subgroup. No difference was found in the rate of serious adverse events between the two subgroups [58]. In 2019, the MENTOR study compared a treatment protocol with RTX and one with cyclosporine in terms of inducing and maintaining a complete or partial remission of proteinuria. The efficacy profile in achieving a complete remission at 12 months proved to be comparable (60% vs. 52%), with RTX having a greater effect in achieving a remission at 24 months (60% vs. 20%) [59]. The Italian study RI-CYCLO, published in 2021, conducted in 74 adults with MN and proteinuria >3.5 g/day, compared a treatment with RTX (1 g) on days 1 and 15 and one with corticosteroids alternating with cyclophosphamide on a monthly basis for 6 months. Patients were randomized into two groups consisting of 37 patients each. After 12 months, 16% of the patients treated with RTX and 32% of the patients treated with the cyclic regimen had achieved complete remission. A total of 62% of patients (23 of 37 patients) receiving RTX and 73% (27 of 37) of those taking the cyclic regimen underwent complete or partial remission. During the 24-month follow-up period, the odds of complete remission and complete or partial remission with RTX were 0.42 (95% CI, 0.26 to 0.62) and 0.83 (95% CI, 0.65 to 0.95), respectively, and 0.43 (95% CI, 0.28 to 0.61) and 0.82 (95% CI, 0.68 to 0.93), respectively, with the cyclic regime. No difference was found in the rate of adverse events between the two subgroups (19% vs. 14%) [60]. The 2021 KDIGO guidelines list several strategies and recommend a fixed dose of 2 g/1 g within 2 weeks, as used in rheumatoid arthritis, or 375 mg/m^2^ administered one to four times weekly as an alternative first-line option for the initial treatment of patients carrying a moderate or high risk of progressive disease. A study including 12 patients with idiopathic MN and NS receiving titrated B-cell treatment was followed with the aim of determining the ideal dosage compared to a group of 24 patients receiving the standard protocol of four weekly doses of 375 mg/m^2^. The results showed that only one patient required a second dose to achieve complete depletion of CD20 cells. After 12 months, the progression of NS manifestations and the proportion of patients achieving disease remission (25%) were identical in both groups. All patients achieved sustained CD20 cell depletion [61]. Another study compared two treatment protocols: NICE, in which patients received two infusions of 1 g RTX every 2 weeks, and the GEMRITUX protocol, with the infusion of two doses of 375 mg one week apart. After 6 months, remissions occurred in 18 (64%) versus 8 (30%) of the NICE and GEMRITUX cohorts. The median time needed to achieve remission was 3 months for the two groups receiving the different protocols. Participants in the NICE cohort had higher circulating RTX levels, lower CD19 counts and lower anti-PLA2R1 antibodies at month 6, demonstrating a greater drug efficacy at higher doses [62]. To confirm these data, Moroni et al. compared two different doses of RTX. One group of 18 patients received a single dose of 375 mg/m^2^ while the second group of 16 patients received two doses. RTX represented the first-line therapy for 56% (*n* = 19) and the second-line therapy for 44%(*n* = 15) patients. Follow up was conducted for 12 months; at the end, 14.7% (*n* = 5) achieved a complete response, 29.4% (*n* = 10) a partial response and 55.8% (*n* = 19) no response. The response occurred approximately 6 months after dosing. The outcome was similar for one and two doses of RTX, with 55.5 and 56% of non-responders respectively [63]. In terms of tolerability and safety, the first studies were conducted on large cohorts of patients with rheumatoid arthritis (RA). In the studies by van Vollenhoven et al., RTX was shown to be generally well tolerated despite the administration of multiple cycles of therapy over an extended period of time. In one study, 3194 patients who received up to 17 cycles of RTX over 9.5 years were compared with a group of 818 patients who received placebo + methotrexate. Rates of serious adverse events and infections generally remained stable over time and across cycles, with a greater tendency for patients to develop low immunoglobulin levels, while serious opportunistic infections were rare. A total of 717 of the patients treated with RTX developed low immunoglobulin IgM levels, and 3.5% (*n* = 112) developed low IgG levels for ≥4 months after more than one cycle [64]. A previous study by Ronal van Vollenhoven et al. demonstrated that most adverse events occur during drug infusion. Out of a total of 2578 RA patients who received at least one course of RTX, 25% developed a reaction during drug administration, but less than 1% of the reactions were classified as serious; rates of adverse events, serious infection and risk of malignancy over time remained stable after each cycle [65]. More recently, in the MENTOR study, serious adverse events were reported in 17% (11 patients of the RTX group and in 31% (*n* = 20) of the CNI group (*p* = 0.06) [59]). Although steroid and cyclophosphamide cyclic therapy is recommended in guidelines for patients carrying a high risk of progressive disease, RTX may be a safer alternative. This was demonstrated in a retrospective observational cohort study comparing the two different therapies and evaluating several endpoints, including all possible adverse events, partial and complete remission of NS and a combination of doubling of serum creatinine, ESRD or death. At a median follow-up of 40 months, there were significantly fewer adverse events in the RTX than in the steroid–cyclophosphamide group (63 vs. 173; *p* < 0.001), both serious (11 vs. 46; *p* < 0.001) and non-serious (52 vs. 127; *p* < 0.001). The cumulative incidence of a first event (35.5% vs. 69.0%; *p* < 0.001) and serious (16.4% vs. 30.2%; *p* = 0.002) or non-serious (23.6% vs. 60.8%; *p* < 0.001) event was significantly lower with RTX [66]. Nevertheless, some issues remain unsolved, especially those regarding the better dosing regimen, long-term safety and efficacy or which strategies to adopt for patients who do not respond to RTX. Monitoring PLA2R antibodies may allow for more personalized treatments and low-dose protocols, with RTX administration given based on both B-cell count and PLA2R Ab levels in order to reduce side effects and costs. Currently, the most reasonable choice is for RTX treatment to be individually tailored to each patient’s disease course. Unfortunately, up to 40% of patients do not respond to rituximab; in addition, hypersensitivity reactions or serum-sick syndrome may occur. Consequently, new anti-CD20 drugs have been developed, namely ofatumumab (OFA) and obinutuzumab (OBI). The first is a humanized monoclonal antibody that binds to CD20 at a different site than that used by rituximab and, moreover, also manages to bind C1q with consequent complement-mediated cytotoxicity. OBI, on the other hand, is another monoclonal antibody which, like OFA, recognizes different epitopes than RTX but which, in addition to complement-mediated mechanisms, uses lysosome-dependent, antibody-mediated and phagocytosis-dependent cytotoxicity mechanisms. Finally, OBI is also able to cause a profound depletion of B cells in the spleen and lymph nodes. While the current data on OFA use come from case reports, two OFA trials evaluating its utilization in MN are ongoing [67].

Table 3 describes the most important studies evaluating RTX use in MN.

## 8. Novel Therapeutic Approaches for Membranous Nephropathy

Immunosuppressive therapy can be associated with serious and potentially fatal side effects. In addition, 20–30% of patients do not achieve a complete or even a partial response to therapy. If response has not been achieved after two or more lines of therapy, alternative therapies may be considered [68]. Findings from the results of studies on the use of adrenocorticotropic hormone (ACTH) have been controversial. In 2005, Berg and Arnadottir published results on a series of 15 patients with MN and NS who achieved complete remission, 14 of whom maintained it during a follow-up period of 14–30 months [69]. In 2006, Ponticelli et al. found no significant difference between two groups of patients with NS and MN treated with the Ponticelli regimen and ACTH, respectively [70]. On the other hand, a prospective, open-label cohort study published by Van de Logt in 2015 showed lower efficacy than cyclophosphamide and the occurrence of several adverse events [71]. In 2019, Kaundal et al. published a study on 11 patients who had previously failed to respond to immunosuppressive therapy and/or RTX. Of the 11 patients, four achieved complete remission and two achieved partial remission, with maintenance of remission at 16 months. Among the adverse events leading to discontinuation of therapy, two patients were reported with fluid accumulation and dyspnea [72]. In each case, the side effects of ACTH were thought to be the same as those of steroids, only to a lesser extent. However, as the evidence for ACTH use is weak, more data are needed. In addition, the mechanism of action is still unclear, although it probably involves melanocortin receptor-1 (MCR-1), which colocalizes with synaptopodin in podocytes. Belimumab, a monoclonal antibody that acts by inhibiting B-cell activating factor (BAFF), is currently used to treat lupus nephritis. In 2020, Barret et al. published an open-label, prospective, single-arm study of 14 patients, 11 of whom discontinued the study, and among which 8 patients achieved partial remission and 1 patient achieved complete remission [73]. Because RTX increases BAFF levels, combination therapy studies have been planned; specifically, a double-blind RCT will compare the efficacy of RTX vs. RTX + belimumab in 124 NM patients [68]. CD19-/CD20-/CD38+/CD138+ plasma cells, which are normally found in the bone marrow and kidney during inflammation, are among the major players in patients who do not respond to CD19- and CD20-depleting agents such as RTX. Bortezomib is able to cause the depletion of plasma cells and is currently used in multiple myeloma therapy. To date, data on the use of bortezomib in MN have come only from case reports, but the results are promising [74,75,76]. Other CD38-targeted agents that may have a role in the treatment of MN include daratumumab and felzartamab. The former has not yet been studied in this context, and the only data are from a single case report [77], while the latter is expected to show results from trials in 2024 [68]. Both lectin and alternative signaling pathways play a role in the pathogenesis of MN. Therefore, complement-targeted therapy will be part of the therapeutic options in the future. Currently, there is only one small study on complement inhibition in MN, and that is with eculizumab, a C5-cleavage inhibitor. However, this study was never published due to negative results, although, as many have pointed out, the dose and duration of this therapy were lower than in protocols used for other diseases [78]. Other molecules that may achieve important results in the future include the factor-B inhibitor iptacopan and pegcetacoplan, targeting C3 and C3b, respectively, and the MASP inhibitor narsoplimab [78]. Since complement inhibition is associated with severe infections by encapsulated organisms, vaccination and prophylaxis against meningococci and pneumococci are essential in all cases. Finally, data on the use of plasmapheresis and immunoadsorption come from case series or case reports in patients with severe disease [79,80].

## 9. Treatment of Secondary Membranous Nephropathy

Secondary MN accounts for almost 25% of MN. It includes forms of disease caused by infections, malignancies, autoimmune diseases and drugs [81]. Recognition of secondary forms is critical because treatment of these forms depends on treatment of the underlying disease and, furthermore, immunosuppressive therapy can be dangerous in malignant or infectious secondary forms. PLA2R antibodies may suggest, but not exclude, primary MN, while THSD7A or NELL1 [23] may ensure malignancy-associated forms [82]; the distribution of IgG subclasses among glomerular deposits may also be helpful, as IgG4 are highly suggestive of primary forms, and IgG1 and IgG2 are found in malignancy-associated disease [83]. The joint use of PLA2R and IgG4 may greatly enhance the ability to distinguish primary from secondary forms [84]. Malignancies most commonly responsible for secondary MN are those of the lung, gastrointestinal tract and prostate and, less frequently, the skin, breast and bladder [85] Resection or treatment of the tumor may lead to the disappearance of NS [86]. In the past, several drugs have been reported to cause MN, including antirheumatic drugs, non-steroidal anti-inflammatory drugs (NSAIDs), penicillamine and captopril. Recently, case reports have described MN caused by gefitinib, a tyrosine kinase inhibitor [87], but not by erlotinib, a drug in the same class. Usually, discontinuation of such therapies results in significant improvement in proteinuria and NS, usually six months or one year thereafter [87]. Recently, several case reports of immune-checkpoint inhibitors have been described [88]. In particular, with regard to RTX administration, the drug could be reinstated after NS remission. In a rare case, the use of siddha drugs have been associated with the occurrence of MN due to the presence of mercury. In such cases, notably in a NELL1 positive patient, significant improvement was achieved by discontinuing such medication [89]. Another case report described the onset of MN in a patient due to sargramostim, a recombinant granulocyte–macrophage colony-stimulating factor (GM-CSF), who recovered completely after discontinuation [90]. Secondary MN may also occur in association with autoimmune liver diseases such as primary sclerosing cholangitis, primary biliary cirrhosis or autoimmune hepatitis. Treatment of these forms is controversial, as liver transplantation has led to significant improvement in proteinuria in some patients, while specific therapy for MN has been required in others, as no improvement had occurred [91]. In the context of RA, secondary MN may be due either to the nephrotoxic effects of disease-modifying antirheumatic drugs (DMARDs), which is the most common or, in the absence of such treatments, it may be related to the disease activity itself. In the first case, albeit with variable timing, MN may improve with the discontinuation of the drugs involved; in the second, much rarer case, a specific therapy for rheumatologic disease involving a combination of steroids, methotrexate and tacrolimus has been shown to be effective in one case report [92]. In cases secondary to ankylosing spondylitis, the use of adalimumab (40 mg/2 weeks), a tumor necrosis factor (TNF)-alpha antagonist, significantly improved proteinuria [93]. Treatment of Grave’s-disease-associated MN has been successful with thiamazole, while others have reported improvement after radioiodine thyroid ablation but not after drug therapy [94,95,96]. However, in patients with myasthenia gravis and MN, treatment options include steroids, ACTH (which has been shown to achieve partial remission), RTX and, finally, thymectomy [97]. If celiac disease is the underlying cause, a trial of a gluten-free diet plus supportive therapy may lead to proteinuria remission, as described by Pestana et al. [98]. Several cases of MN secondary to tuberculosis agents, such as *Mycobacterium tuberculosis* and the much rarer *Mycobacterium shimoidei,* have been described without overt renal tuberculosis. Also, in this case, the initiation of pathogen-specific antibiotic therapy against these mycobacteria, namely clarithromycin, rifampicin and ethambutol, can significantly improve proteinuria and the renal status [99]. In hepatitis-related MN, immunosuppressive therapy may increase viral replication. Specific treatment of the infection, including the use of entecavir, plus plasmapheresis and steroids, has been shown to improve both th viral load and NS [100]. Still, a rare cause of secondary MN may be syphilis. It is important to detect luetic MN, as the use of steroid therapy may worsen the symptoms and lead to an advancement in the stage of the underlying disease, while the use of specific therapy against the pathogen, *Treponema pallidum*, may allow for the rapid improvement of the NS and avoid the use of steroid therapy. Intramuscular benzathine penicillin once a week for three weeks has been shown to be effective [101,102]. In addition, MN may occur during chronic graft-versus-host disease (CGVHD) in patients who have undergone bone marrow transplantation. In such cases, RTX has been used, which is both safe and effective [103]. If paroxysmal nocturnal hemoglobinuria is superimposed, complement-directed therapy may be helpful [104]. In addition, secondary MN of Castleman’s disease has been successfully treated with tocilizumab from both a renal and hematological perspective [105,106]. Another cause of MN is represented by chronic lymphocytic leukemia; such cases have been treated with RTX over the years and, more recently, venetoclax has been successfully used in a refractory patient [107]. Membranous lupus nephritis accounts for almost 15% of lupus nephritis (LN). It is associated with a low risk of progression to ESRD but, conversely, carries an increased risk of thromboembolic complications and is often complicated by nephrotic-range proteinuria and its clinical manifestations. Mycophenolate mofetil is recommended as an initial treatment, but other studies have demonstrated the efficacy of tacrolimus. Alternative therapies include cyclosporine and cyclophosphamide, while corticosteroids alone do not induce remission [108]. However, when proliferative classes are superimposed on lupus nephritis, the treatment may change significantly [82]. Finally, MN is the most prevalent glomerulonephritis associated with sarcoidosis. Although the mechanism by which renal injury develops in patients with sarcoidosis is not known, there is ample evidence that these patients respond to steroids alone, unlike patients with primary MN [109].

## 10. Membranous Nephropathy in Children

MN is a rare disease in children. It accounts for less than 2% of biopsies in American children [110], although its prevalence is increasing year by year and has reached 6% in Chinese children [111]. Several studies have shown that its prevalence is higher in the adolescent subgroup than in children (18.5% in adolescents compared with 3% in children in a retrospective analysis in Pakistan [112] and 9% compared with 3% in a cross-sectional study of biopsies in China [111]). However, it is not possible to determine the exact prevalence of the disease as MN is a diagnosis of exclusion, and patients with NS are only biopsied only if they do not respond to steroids, while patients with hematuria, a more common feature of the disease than in adults, are not commonly biopsied [113]. Most of the available data are either monocentric or have been extrapolated from national registries. In the pediatric population, secondary MN is more common than primary MN, in which systemic causes such as systemic lupus erythematosus (SLE) or hepatitis play a role. Compared with adult patients, hematuria is more common in children, while hypertension, proteinuria or a decrease in kidney function are less frequent. Stage I is also more common in children by electronic microscopy. In addition, PLA2R antibodies were more common in adults than in children, and when the pediatric population was divided into subgroups, adolescent patients were more frequently affected than younger ones [114]. A retrospective analysis of 217 cases of primary MN, spanning a 10-year period from 2008 to 2017, found that the most common manifestation was NS (59.9%) and that the subsequent edema had a more insidious onset than minimal-change disease or focal segmental glomerulosclerosis. Because of the low prevalence of the disease, there is a little prognostic evidence; however, in this study, the 5-year and 10-year survival was 95.3% and 67.8%, respectively, with hypertension and a proteinuria greater than 50 mg/kg/day as risk factors for a worse renal outcome [115]. In children, searching for specific autoantibodies can also improve both diagnosis and treatment. Unlike in the adult population, there is limited data on the role of PLA2R, while THSD7A antibodies have not been studied in patients under 18 years of age. Zhang et al. found a prevalence of 42.1% PLA2R-positive antibodies in 38 patients with a diagnosis of primary MN in a Chinese retrospective study [114], while another single-center report in South Asia reported a rate of 70% [116]. In addition, other responsible antigens have been described, such as the neutral endopeptidase protein, involved in the fetomaternal alloimmune form, and the cationic form of bovine serum albumin [117]. Recently, the Sema3b antigen was discovered, which seems to be most correlated with the pediatric population and is responsible for 15% of pediatric cases of MN in children, especially in patients younger than two years. However, there is little therapeutic and prognostic evidence for Sema3b-positive patients due to a lack of cohort studies. Some patients recovered spontaneously, while others required immunosuppressive therapy [13]. Dettmar et al. studied 12 children with MN and concluded that pediatric patients in whom PLA2R antibodies have disappeared can only be safely treated with supportive therapy, just like the adult population [117] Interestingly, two cases of double positivity for both PLA2R and Sema3b have also been reported, showing that positivity for individual antigens is not mutually exclusive [118]. While secondary forms of MN require specific treatment for the underlying disease, there is currently no evidence-based standard therapy for primary membranous nephropathy in children. A renal biopsy is not usually performed in patients with steroid-sensitive nephrotic syndrome. About 30% of patients can achieve remission without drug therapy, while the rest require specific treatment. Conservative treatment alone can be used if proteinuria is not in the nephrotic range; otherwise, the risk of progression to end-stage renal disease warrants more targeted treatment [119]. As Valentini described in a retrospective study, 50% of patients with nephrotic pMN syndrome do not respond to steroid therapy, while the remainder experience complete (10%) or partial (40%) remission [120]. Data on the treatment of immunosuppression in pediatric patients come from case reports or case series. The lack of RCTs on the pediatric population makes it difficult to choose one immunosuppressant over another. Valentini’s study found a 75% response rate to a 3-month course of cyclophosphamide (2 mg/kg/day) with steroids given daily [120]. Lee et al. used cyclosporin with a response rate of 100% after 6 months but with relapse after discontinuation of the drug [121], while Chen found no difference between cyclosporin and tacrolimus [122]. Bhimma et al. achieved partial remission with MMF (1200 mg/m^2^) [123]. A report from a single center in South Asia investigated the response to various immunosuppressive therapies in 48 patients with biopsy-proven pMN, such as cyclical CYC/GC, CNI/GC, rituximab, MMF, azathioprine and prednisone alone, with no significant difference in outcomes [116]. In this study, all cases with resistant disease responded to rituximab administration with either complete or partial remission, and the response rate in resistant patients was even better than in patients treated with first-line therapy. Currently, there are no recommendations on the use of rituximab as a first-line agent, but the ever-growing number of case reports and case series reporting excellent results make it a promising drug for the of the disease in pediatric patients also. In addition, the ideal dosage for these patients has not yet been established; the use of 1 g every 2 weeks as well as 375 mg/m^2^ once a week for 4 weeks have been reported. In addition, in the case of rituximab-resistant disease or in patients suffering from rituximab serum sickness [124], new anti-C20 antibodies such as ofatumumab, obinutuzumab and ocrelizumab could be considered [125]. Finally, there are encouraging data from preliminary studies on the use of probiotics. Yamaguchi et al. found that daily administration of *Clostridium butyricum* preparations reduced the recurrence of nephrotic syndrome compared with patients treated with placebo and that, in the same group, fewer patients received rituximab for recurrences [126].

## 11. Membranous Nephropathy in Pregnancy

During pregnancy, idiopathic MN is a rare cause of NS; there are few case reports in the literature [127]. In women of child-bearing age, MN is often secondary to other conditions, such as systemic lupus erythematosus, hepatitis B or hepatitis C viral infections and syphilis, and in association with drug exposure (especially biologic agents and NSAIDs) or, less commonly, with neoplastic disease [128]. The absence of risk factors, such as NS, reduces the impact of this pathology on the occurrence of maternofetal complications [129]. The cohort studied by Peckham in 1987 had the highest number of serious outcomes: fetal loss in 24% of cases, preterm birth in 43%, CKD progression in 9%, the onset of hypertension in 46% of patients and significant proteinuria in 54.5% [130]. Liu et al., in their most recent retrospective study performed on 27 pregnancies from 2008 to 2018, highlighted the occurrence of maternal–fetal adverse events in 10 of these cases, including fetal loss (11%), preterm delivery (26%) and severe pre-eclampsia (15%). Severe hypoalbuminemia, severe proteinuria (especially before 20 weeks of gestation), positive anti-PLA2R and no remission were risk factors for worse outcomes [131]. Treatment of this condition during pregnancy is challenging. Due to their teratogenic effects, ACE inhibitors, lipid-lowering agents, warfarin, mycophenolic acid and cyclophosphamide are contraindicated. Alpha-2 adrenergic receptor blockers, such as methyldopa, and calcium blockers, like long-acting nifedipine or labetalol, are the first-line drugs in maintaining blood pressure [132]. Because of the likely occurrence of adverse events in newborns, such as hypotension and, often, transient bradycardia and hypoglycemia, other beta-blockers are used in the second line of treatment. Diuretics are not usually used in pregnancy; however, these may be administered in NS refractory to immunosuppressive drugs but under medical supervision to avoid pre-renal acute kidney injury (AKI) forms. Corticosteroids, such as prednisone and methylprednisolone, and calcineurin inhibitors, such as tacrolimus and cyclosporine, are the first-line treatment option [133]. In the last trimester of pregnancy, RTX should be avoided for the possible B-cell depletion in the newborn [134].

## 12. Membranous Nephropathy in Kidney Transplant Recipients

In kidney transplant recipients MN can occur either as a recurrent disease, when the diagnosis of MN has already been made in native kidneys, or as de novo MN (DNMN), when the cause of kidney failure is a different disease. It is not always possible to distinguish between these two identities because the cause of the primary failure may be unknown, in which case other authors use the term transplant MN [135]. Beyond this division, however, MN increases the risk of graft failure [136]. Table 4 shows the difference between de novo membranous nephropathy and recurrent membranous nephropathy.

Determining the exact incidence of MN is difficult because indications for performing biopsies differ between centers; in addition, not all centers perform electron microscopy and immunofluorescence routinely. However, recurrent membranous nephropathy (RMN) can occur in up to 40% of cases, while DNMN is reported in up to 2% in the adult population and up to 9% in the pediatric population [137]. Of note, the latter is more frequent in patients with HCV infection [138], CMV infection [139] Alport syndrome or kidney obstruction or in association with IgA nephropathy [140]. Moreover, the risk of MN recurrence is even higher in the case of a second transplant [141] and correlates with the anti-PLA2R antibodies titer [142]. On the other hand, association between the PLA2R antibodies titer prior to transplant and the recurrence of membranous nephropathy was statistically significant in a cohort of 63 biopsy-proven RMNs [142]. Berchtold et al. studied 105 kidney transplant recipients with MN and matched donors and found an association with single-nucleotide polymorphisms in HLA-DRB1 and HLA-DQA1 and three SNPs in PLA2R1 [143]. These results suggest that further evaluation in living-related-donor transplants may be needed. In addition, DNM is associated with IgG1 autoantibodies, unlike MN in native kidneys and in RMN where the most prevalent subclass is IgG4 [144]. Compared with patients with RMN, patients with DMN have a higher risk of antibody-mediated rejection and worse graft survival [145]. KDIGO 2021 underlines the pivotal role of PLA2R measurement. If primary MN is not associated with PLA2R, they suggest a monthly evaluation of proteinuria for at least six months after transplantation and to perform a biopsy if this exceeds 1g per day. If, on the other hand, the primary MN is PLA2R positive, they recommend PLA2R monitoring with a frequency depending upon the original titer of antibodies. An increase in such measurements can anticipate a relapse of the disease, anticipating the possibility of a biopsy even if the proteinuria is below 1g per day. If the proteinuria exceeds 1 g, Grupper et al. [146] suggest an RTX infusion, with 1 g given two weeks apart. Regardless of the severity of the proteinuria, RAAS blockade and a check of immunosuppressive regimen adherence is strongly recommended [7]. In patients who do not achieve immunological remission, i.e., with detectable PLA2R antibodies, further administration of RTX may be required [147]. In RMN, it has been used successfully, with 75% of patients having complete or partial remission at 1 year [148]. Although this has not been evaluated, it is reasonable to think that a similar evaluation could apply to other antibodies such as anti-THSD7A [7]. If a secondary cause such as infection or a malignancy is identified, treatment of the latter may resolve the MN [149]. In some cases, only increasing tacrolimus serum levels and the use of RAAS blockade improved proteinuria and kept the renal function stable [149]. In addition, a case of post-transplant NELL-1-positive MN resulting in NS has been reported that was managed only by adding a double RAAS blockade, atorvastatin and torsemide, which resulted in partial remission of proteinuria and subsequent NELL-1 antibodies disappearance [150]. Another case described a recurrence of MN positive for anti-semaphorin 3B antibodies in a 7-year-old child who responded to RTX and enalapril with a marked improvement in proteinuria and complete remission within 4 months [151]. Thus, if these antibodies are present at the time of transplantation, they may help to predict the recurrence of MN.

## 13. Economic Considerations and Patients’ Preference

Another important issue concerns the costs of such therapies; in fact, it is essential to evaluate the impact that these treatments have on the healthcare budget [152]. Also, cost evaluations should take into account not only the drugs themselves but also various factors including the treatment for adverse events, blood drug-concentration monitoring and end-of-hospitalization laboratory examination. At the moment, few studies have carried out a cost-effective analysis on the treatment of membranous nephropathy. In 2018, Hamilton et al. compared the modified Ponticelli regimen to rituximab; despite the high single-dose cost of the latter, at five years post treatment, it remained the cheapest option in the short and medium term [153]. According to a 2020 Chinese meta-analysis, in China, the least expensive treatment was cyclophosphamide, while the most expensive was rituximab in both UK and China. Also, MMF and tacrolimus was characterized by high cost [154]. Conversely, in 2023, Xu et al. found out that cyclophosphamide and rituximab were comparable from a cost-effectiveness point of view, while a negative incremental cost-effective ratio was underlined for calcineurin inhibitors [152]. However, the use of biosimilars may help in making rituximab more affordable [155].

Moreover, the modified Ponticelli regimen requires various hospital admissions to perform steroid and cyclophosphamide infusions, while RTX can be administered in an outpatient setting.

To the best of our knowledge, currently no studies have systematically evaluated patients’ choice over the various therapeutical opportunities proposed for MN. In our experience, CYC raises concern due to the risk of malignancy and the effect on fertility; similarly, other patients wish to avoid steroids or CNI administration for their side effects. Rituximab, conversely, appears to be well tolerated, especially when administered in an outpatient setting.

## 14. Conclusions

In this review, we have summarized the most important points on the treatment of MN. Although the discovery of new autoantibodies may allow for diagnosis without a renal biopsy and provide important prognostic and therapeutic clues, currently, up to 30% of patients do not respond to therapy despite supportive care and appropriate immunosuppressive therapy, leading to ESRD. The use of new drugs targeting plasma cells and the complement system, which are currently under investigation, could help to significantly reduce this percentage. On the other hand, further studies on specific contexts such as childhood, pregnancy and kidney transplantation are needed to improve diagnosis and to choose the most appropriate treatment.

## Figures and Tables

**Table 1:** Different antigens and their features.

Autoantibody	Year	Protein	Association	Positivity Rate	IgG Subtype	Circulating Ab
PLA2R	2009	Transmembrane protein	pMN	70% of pMNs	IgG4	Yes
THSD7A	2014	Transmembrane protein	Malignancy	5% of all MNs	IgG4	Yes
Exostosin 1-2	2019	Glycosyl transferase	MLN	50% of cases with class V	IgG1	No
NELL-1	2020	Protein kinase C-binding protein	Malignancy	10% of all MNs	IgG1	Yes
SEMA-3b	2020	Transmembrane protein	Family history	15% of pediatric cases	IgG1	Yes
Contactin-1	2021	Neural cell glycoprotein	Autoimmune neuropathy	80% of autoimmune neuropathies	IgG4	Yes
PCDH7	2021	Transmembrane protein	Prostate carcinoma	5.7% of PLA2R − cases	IgG1/4	Yes
HTRA1	2021	Serine protease	pMN	4.2% of pMNs	IgG4	Yes
NCAM	2021	Immunoglobulin proteins	LES	6.6% of MLNs	Unknown	Yes
TGFBR3	2021	Transmembrane protein	LES	6% of MLNs	IgG1/2/3	No
FAT1	2022	Transmembrane protein	HSCT	83% of HSCT MNs	IgG4	Yes
Netrin G1	2022	Secreted glycoprotein	pMN	0.003% of MNs	IgG4	Yes

Ab, antibody; FAT1, protocadherin FAT1; HSCT, hematopoietic stem cell transplantation; HTRA1, HtrA serine peptidase 1; MN, membranous nephropathy; MLN, membranous lupus nephritis; NCAM, neural cell adhesion molecule; NELL-1, neural-tissue-encoding protein with EGF-like repeats-1; pMN, primary membranous nephropathy; PLA2R, phospholipase A2 receptor 1; PCDH7, protocadherin 7; THSD7A, thrombospondin-1-domain-containing 7 A; TGFRB3, transforming growth factor beta receptor 3; SEMA-3b, semaphorin-3b.

**Table 2:** Different protocols described in KDIGO 2021.

Drug	Protocol
CYCLICAL CYCLOPHOSPHAMIDE	Methylprednisolone 1 g for 3 days at months 1, 3, 5
	Prednisone 0.5 mg/kg/d at months 1, 3, 5
	Cyclophosphamide 2.5 mg/kg/d at months 2, 4, 6
CONTINUOUS CYCLOPHOSPHAMIDE	Methylprednisolone 1g for 3 days at months 1, 3, 5
	Prednisone 0.5 mg/kg/d every other day for 6 months and subsequent tapering Cyclophosphamide 1.5 mg/kg/day continuously for 6 months
RITUXIMAB	Rituximab 1 g twice two weeks apart or 375 mg/m^2^ up to 4 times at weekly intervals
TACROLIMUS	Tacrolimus 0.05–0.1 mg/kg/d. Plasma level: 3–8 ng/mL
CYCLOSPORINE	Cyclosporine 3.5 mg/kg/g. Plasma level: 125–225 ng/mL

**Table 3:** Most important studies involving the use of RTX in MN.

	GEMRITUX (2017)	MENTOR (2019)	STARMEN (2020)	RICYCLO (2021)
Patients (n)	75	130	86	74
RTX group	RTX 375 mg/m^2^ on days 1–8 + Supportive therapy	RTX 1g on days 1–15 + RTX second course at 6 months if no complete remission + Supportive therapy	Oral tacrolimus (blood levels 5–7 ng/mL) for 6 months + RTX 1 g at day 180 with tacrolimus tapering and complete withdrawal at month 9	RTX 1g on days 1–15 + Supportive therapy
Control group	Supportive therapy	Cyclosporine (blood target levels 125 to 175 ng/mL) and tapering after 6 months if remission achieved + Supportive therapy	Methylprednisolone 1 g × 3 (months 1, 3, 5) and oral prednisone (0.5 mg/kg/day) for the following 27 days + Oral cyclophosphamide (1–2 mg/kg/days) at months 2, 4, 6	Methylprednisolone 1 g × 3 (months 1–3–5) and oral prednisone (0.5 mg/kg/day) for the following 27 days + Oral cyclophosphamide (2 mg/kg/days) at months 2, 4, 6
Primary endpoint	Complete or partial remission of proteinuria after 6 months	Complete or partial remission of proteinuria after 24 months	Complete or partial remission of proteinuria after 24 months	Complete or partial remission of proteinuria after 12 months
Primary outcome	RTX *n* = 13 (35.1%) vs. control group *n* = 8 (21.1%) *p* = 0.21	RTX *n* = 39 (60%) vs. control group *n* = 13 (20%) *p* < 0.001	RTX group *n* = 25 (58.1%) vs. control group *n* = 36 (83.7%)	RTX group *n* = 23 (62%) vs. control group *n* = 27 (73%)
PLA2R antibodies trend	RTX group = 50% of deletion after 6 months vs. control group = 12% *p* = 0.004	Better reduction in RTX group compared with control group	Significant reduction in both groups. Higher immunological response at months 3 and 6 in control group compared with RTX group (month 3: 77% vs. 45; month 6: 92% vs. 70%)	Reduction in both groups during follow up but faster decrease in RTX group
Adverse events	Serious events in 21% of both groups	Serious events in 11 patients in RTX group (17%) vs. 20 in control group (31%) *p* = 0.04	More adverse events in control group (19%) than in RTX group (14%) *p* = 0.04	Serious adverse events in 19% in RTX group vs. 14% in control group

**Table 4:** Main differences between de novo membranous nephropathy and recurrent membranous nephropathy.

DE NOVO MEMBRANOUS NEPHROPATHY	RECURRENT MEMBRANOUS NEPHROPATHY
Different cause of native kidney failure	MN diagnosis already made in native kidneys
Prevalence: 2% in adult; 9% in pediatric	40% of the cases
Prevalently IgG1 autoantibodies	Prevalently IgG4 autoantibodies
Higher risk of antibody-mediated rejection	Lower risk of antibody-mediated rejection compared with de novo membranous nephropathy
Increase immunosuppressive therapy or consider plasmapheresis	Immunosuppressive therapy may cause the disappearance of antibodies
Rituximab or cyclophosphamide for worsening disease	Rituximab for worsening disease
Worse graft survival	Better graft survival

MN, membranous nephropathy; DNMN, de novo membranous nephropathy.

## Data Availability

Not applicable.
